# Levels of Human Erythrocyte Membrane-Bound and Cytosolic Glycohydrolases Are Associated with Oxidative Stress in Erectile Dysfunction Patients

**DOI:** 10.1155/2014/485917

**Published:** 2014-08-05

**Authors:** L. Massaccesi, G. V. Melzi d'Eril, G. M. Colpi, G. Tettamanti, G. Goi, A. Barassi

**Affiliations:** ^1^Department of Biomedical, Surgical and Dental Sciences, University of Milan, School of Medicine, Via Saldini 50, 20133 Milan, Italy; ^2^Department of Health's Sciences, University of Milan, Milan, Italy; ^3^U.O. Andrology, San Paolo University Hospital, Milan, Italy; ^4^IRCCS Policlinico San Donato, San Donato Milanese, Italy

## Abstract

Oxidative stress (OS) and production of NO, by endothelium nitric oxide synthetase (eNOS), are involved in the pathophysiology of erectile dysfunction (ED). Moreover, OS induces modifications of the physicochemical properties of erythrocyte (RBC) plasma membranes and of the enzyme content of the same membranes. Due to their role in signalling early membrane alterations in OS-related pathologies, several plasma membrane and cytosolic glycohydrolases of human RBC have been proposed as new markers of cellular OS. In RBC, NOS can be activated and deactivated by phosphorylation/glycosylation. In this regulatory mechanism O-*β*-N-AcetylGlucosaminidase is a key enzyme. Cellular levels of O-GlcNAcylated proteins are related to OS; consequently dysfunctional eNOS O-GlcNAcylation seems to have a crucial role in ED. To elucidate the possible association between RBC glycohydrolases and OS, plasma hydroperoxides and antioxidant total defenses (Lag-time), cytosolic O-*β*-N-AcetylGlucosaminidase, cytosolic and membrane Hexosaminidase, membrane *β*-D-Glucuronidase, and *α*-D-Glucosidase have been studied in 39 ED patients and 30 controls. In ED subjects hydroperoxides and plasma membrane glycohydrolases activities are significantly increased whereas Lag-time values and cytosolic glycohydrolases activities are significantly decreased. These data confirm the strong OS status in ED patients, the role of the studied glycohydrolases as early OS biomarker and suggest their possible use as specific marker of ED patients, particularly in those undergoing nutritional/pharmacological antioxidant therapy.

## 1. Introduction

Erectile dysfunction (ED) [1] is defined as the inability to achieve or maintain erection of the penis during sexual intercourse. Although ED is a multifactorial process, vascular disease of the penile arteries is an important cause in up to 80% of cases [2]. Penile erection results from the interaction of different physiologic systems involving the central and peripheral nervous system and endothelial and vascular smooth muscle cells of the corpora cavernosa [3]. There is growing interest among researchers in the role of oxidative stress (OS) in the pathophysiological mechanism of ED [4]. The impairment of penile vascular function is associated with erectile dysfunction in a variety of vascular disorders characterized by a strong oxidative stress, including diabetes mellitus [5]. OS is the direct consequence of imbalance between the production of reactive oxygen/reactive nitrogen species (ROS and RNS) and intracellular antioxidant defenses.

It is well known that OS leads to alterations of physicochemical properties and enzyme activities [6] of erythrocyte plasma membrane.

Glycohydrolases catalyse the hydrolysis of specific glycosidic linkages in naturally occurring glycosides or glycoconjugates. They are ubiquitously distributed, mainly located in lysosomes, and have been found in other intracellular vesicles with acidic matrix, in plasma membranes, cytosol, and blood plasma [7, 8]. Plasma membrane and cytosol glycohydrolases, namely, Hexosaminidase (HEX), *β*-D-Glucuronidase (GCR), *α*-D-Glucosidase (*α*-GLU), and O-*β*-N-Acetyl-glucosaminidase (O-GlcNAcase,), were also found in human erythrocytes where [9, 10] they have a role in signalling early membrane alterations [6], in pathologies related to strong oxidative stress, such as type 2 diabetes mellitus [11] or Down's syndrome [12, 13]. For these reasons their use as new markers of cellular oxidative stress has been suggested [6, 13, 14].

In particular, O-GlcNAcase is a nuclear-cytoplasmic enzyme, recently found also in erythrocyte plasma membrane [10], cloned and characterized for the first time by Dong and Hart [15]. Together with O-GlcNAc transferase (OGT), O-GlcNAc ase plays a key role in the dynamic process of the O-linked *β*-N-acetylglucosamine (O-GlcNAc) proteins [16]. A large group of cytosolic and nuclear proteins carry single residues of O-linked GlcNAc and undergo functional modification after insertion or detachment of GlcNAc [17]. In such contest, O-GlcNAcase has a crucial role in the regulation of NO production (NO is, probably, the principal neuromodulator of penile erection [18]) acting in the regulatory cycle of O-glycosylation/phosphorylation of the specific residue of Ser 1177 in the active site of endothelium nitric oxide synthetase (eNOS).

A recent study has shown that in human erythrocytes (RBC) a NOS (RBC-NOS) is present which, like eNOS, can be phosphorylated/glycosylated at the same specific site [19] and that its activation is induced by fluid shear stress stimuli and vascular endothelium growth factor signalling. Consequently, and considering the role of RBC-NOS in NO production [20] and the recently proposed role of RBC-NOS in releasing NO during shear stress [21, 22], it is possible to hypothesize that also RBC-NOS could have a role in cavernous smooth muscle relaxation. Furthermore, recent studies show an impairment of NO pathway in RBC associated with a decrease of NO synthase activity in pathologies related to strong oxidative stress as, for example, coronary artery disease [23, 24].

Therefore we measured the above-mentioned enzyme activities both in membrane and cytosol of erythrocytes of ED patients to evaluate their possible involvement in ED and their possible use to mark oxidative stress and to monitor the patients that undergo antioxidant treatment [25].

## 2. Materials and Methods

### 2.1. Subjects

39 ED patients, aged 52.9 ± 10.6, were recruited from* U.O. Andrology of San Paolo University Hospital, Milan, Italy*. Controls were 30 adult male volunteer blood donors, aged 50.1 ± 5.9, from the Italian association of blood volunteers (AVIS) in Milan, Italy. This investigation conforms to the principles outlined in the Declaration of Helsinki. Signed informed written consent was obtained from all subjects before their participation in the study. No ethics approval was required because no additional blood was needed for this study and this was explained thoroughly to all patients. This procedure has been accepted by several journals [26].

Erectile function was assessed on an appropriate clinical work-up study and by using the abridged five-item version of the International Index of Erectile Function questionnaire (IIEF-5), a validated, self-administered questionnaire [27]; all subjects were classified with erectile dysfunction (IIEF = 13 ± 5). All subjects were selected based on the following exclusion criteria: coronary artery disease, diabetes mellitus, hypertension, malignancy, renal failure, congestive heart failure, systemic inflammatory disease, or heart arrhythmias.

### 2.2. Materials

Commercial chemicals were of the highest available grade. The water routinely used was freshly redistilled in a glass apparatus. Bovine serum albumin (BSA), CuSO_4_, 1,6-Diphenyl-1,3,5-hexatriene (DPH), its cationic derivative 1-[4-(trimethyl-amino)-phenyl]-6-phenyl-1,3,5-hexatriene (TMA-DPH), 4-Methylumbelliferone (MUB), *α*- and *β*-glycosides, used as enzyme substrates, and N-Acetyl-D-galactosamine (GalNAc) were purchased from Sigma Chemical Co. (St. Louis, MO,USA). MUB, purchased from Fluka GmbH (Buchs, Switzerland), was recrystallized from ethanol three times.

d-ROMs kit test was purchased from Diacron International (Grosseto, Italy).

### 2.3. Blood Sampling and Preparation of Erythrocyte Membrane and Cytosolic Fractions

Erythrocytes and plasma were prepared from heparinized venous blood. In brief, after collection, the blood samples were immediately centrifuged for 15 min at 3000 ×g and plasma immediately withdrawn and stored at −20°C until further assays. The buffy coat, aspirated from the surface of the pellet, was discarded and the residual material was diluted (1 : 1, v/v) with phosphate buffer solution (PBS) at pH 7.4 and filtered with Leucostop 4LT-B filters (Baxter Spa, Mirandola, Modena, Italy) in order to remove all platelet and leukocyte contaminants. The filtered material, containing only erythrocytes, was centrifuged for 5 min at 1200 ×g and the pellet was washed twice (1200 ×g per 5 min) with PBS at pH 7.4 and immediately used for ghost preparation. Unsealed ghost membranes were prepared at 4°C according to the method of Steck and Kant [28], which employs hypotonic treatment (from 5.0 to 1.25 mmol/L PBS, pH 8.0). Cytosol was obtained by pooling the three supernatants from the hypotonic treatments for the preparation of unsealed ghost membranes.

### 2.4. Enzyme Assays

The following glycohydrolases were assayed with a microfluorimetric method utilizing 4-MU-glycosides as substrates and following the methods of Goi et al. [9]: O-GlcNAcase (E.C. 3.2.1.169), HEX (E.C. 3.2.1.52), GCR (E.C. 3.2.1.31), and *α*-GLU (E.C. 3.2.1.20). Briefly, 50 *μ*L of erythrocytes plasma membrane or cytosolic fractions, diluted as necessary, was incubated in a final volume of 250 *μ*L containing 25 *μ*L of 50 mmol/L citric acid-sodium phosphate buffer at appropriate pH, and 175 *μ*L of the specific substrate dissolved in water. The mixtures were incubated in a shaker bath at 37°C for the established period of time. The reaction was stopped and fluorescence developed by adding 750 *μ*L of alkaline solution (0.2 mol/L glycine-NaOH buffer, containing 0.125 mol/L NaCl, pH 10.75). The control incubation mixtures (blanks) were set up using incubation mixtures without the erythrocyte sample, which were incubated separately and added immediately before stopping the reaction. To determine the activity of O-GlcNAcase, which is only active toward GlcNAc derivatives, the assay employed 4-MUB-N-acetyl-*β*-D-glucosaminide as substrate, in the presence of GalNAc (50 mmol/L) as a competitive inhibitor of hexosaminidase A and hexosaminidase B [29]. Enzyme activities in plasma membrane and cytosol are expressed as *μ*U/mg of protein and mU/mL, respectively. Protein content was determined by the method of Lowry et al. [30], using crystalline bovine serum albumin as the standard.

### 2.5. Plasma Membrane Fluorescence Anisotropy

We assessed the membrane fluidity of hydrocarbon core and of the region of phospholipid head groups by measuring, respectively, the steady-state anisotropy of DPH and TMA-DPH by Jasko FP-770 spectrofluorimeter, following the method of Cazzola et al. [31]. The DPH and TMA-DPH probes were excited at a wavelength of 340 nm, and the emission wavelength was set at 420 nm. Samples were then excited with vertically polarized light and we measured the intensity of emitted light, vertically (Iv) and horizontally (Ih) polarized with respect to the exciting light. Anisotropy was calculated using the equation *rs* = Iv − Ih/Iv + 2Ih, where *rs* is inversely related to membrane fluidity.

### 2.6. Evaluation of Plasma Peroxidation and Plasma Oxidative Balance

Plasma lipid hydroperoxide levels (ROS) were determined colorimetrically according to Trotti et al. [32] and expressed as H_2_O_2_ equivalents.

The kinetics of plasma oxidation, induced by addition of 0.5 M CuSO_4_, were determined at 37°C by monitoring the development of fluorescence at 430 nm, setting the excitation at 355 nm as described by Cervato et al. [33] by Multilabel Counter Wallac 1420 from PerkinElmer. This method allows the evaluation of the peroxidation kinetics monitored following the formation of fluorescent adducts originating from the reaction of aldehydes (derived from lipid peroxidation promoted by Cu^++^ bound to apolipoproteins) with amino groups of plasma proteins and/or phospholipids. The kinetic is expressed by a sigmoid curve that can be divided into an initial latency phase, followed by a second propagation phase. The initial latency phase (Lag-time, expressed in minutes and calculated as the intercept of the linear regression of the propagation phase with that of the latency phase) is an index of lipoproteins resistance to peroxidation.

### 2.7. Statistical Analysis

Test of Kolmogorov-Smirnov showed no significant differences from the normal distribution of data. Therefore parametric techniques were used. Means were compared by one-way analysis of variance (ANOVA). All analyses were performed using the SPSS STATISTIC 22 package (SPSS Inc., Chicago, IL, USA).

## 3. Results

The clinical and metabolic parameters of the subjects in study are reported in Table 1. All the parameters evaluated fall within the reference ranges, both in controls and in ED subjects.

**Figure 1:**
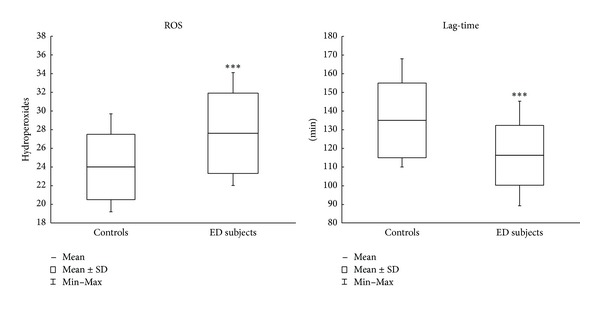
Plasma peroxidation parameters. Hydroperoxides are expressed as equivalent of H_2_O_2_ mg/dL of plasma. ***P < 0.001 controls versus ED subjects.

Plasma hydroperoxide levels and plasma susceptibility to peroxidation (Lag-time) are reported in Figure 1. As expected, hydroperoxide levels of ED patients (mean ± SD: 27.6 ± 4.3; (range: 20.2–36.0)) are significantly (*P* < 0.001) higher (+15%) than the control group (24.0 ± 3.5; (19.2–29.7)). The time lapse necessary to the total antioxidant defense system of the plasma to inhibit the peroxidative process (Lag-time) is significantly (*P* < 0.001) lower (−15%) in ED patients (116 ± 16; (89–145)) than in the control group (135 ± 20; (110–168)).

**Figure 2:**
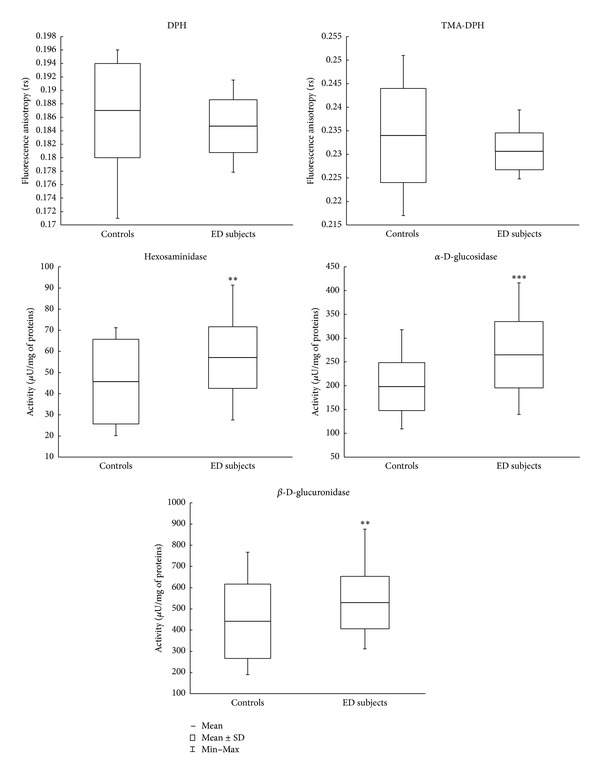
Erythrocyte membrane anisotropy and membrane-bound glycohydrolases activities. DPH: Diphenyl-1,3,5-hexatriene. TMA-DPH: 1-[4-(trimethyl-amino)-phenyl]-6-phenyl-1,3,5-hexatriene. **P < 0.01 and ***P < 0.001 controls versus ED subjects.

In Figure 2 the analyses of fluorescence anisotropy (*rs*) of the erythrocyte membrane are reported. DPH probe shows no significant differences between ED patients (0.185 ± 0.004; (0.178–0.192)) and control group (0.187 ± 0.007; (0.171–0.196)); a similar pattern is followed by TMA-DPH probe with no significant differences between ED patients (0.231 ± 0.004; (0.225–0.240)) and control group (0.234 ± 0.010; (0.217–0.251)). Instead the enzymatic activities of membrane bound glycohydrolases, Figure 2, are significantly higher in ED than in controls: HEX *P* < 0.01 (+27%) (ED patients: 58.1 ± 14.9; (27.9–93.1); control group: 45.7 ± 20.0; (19.8–71.2)); GCR *P* < 0.01 (+22%) (ED patients: 539.3 ± 126.7; (315.8–893.7); control group: 441.7 ± 175.1; (190.2–768.6)); and *α*-GLU *P* < 0.001 (+36%) (ED patients: 269.8 ± 71.2; (141.4–424.4); control group: 198.3 ± 50.3; (109.7–317.8)).

**Figure 3:**
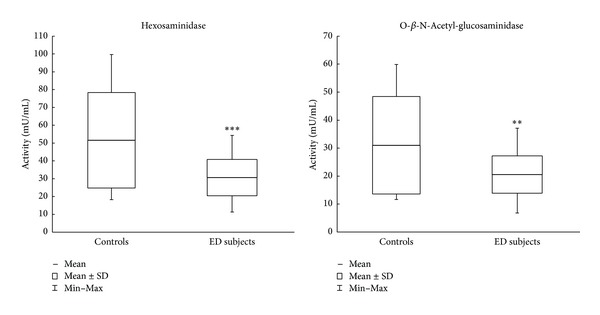
Glycohydrolases activities in the erythrocyte cytosol. **P < 0.01 and ***P < 0.001 controls versus ED subjects.

Cytosolic HEX and O-GlcNAcase activities, showed in Figure 3, are significantly lower in ED patients than in controls: HEX *P* < 0.001 (−39%) (ED patients: 31.3 ± 10.4; (11.5–55.6); control group: 51.6 ± 26.8; (18.3–99.7)) and O-GlcNAcase *P* < 0.01 (−33%) (ED patients: 21.0 ± 6.8; (6.9–37.9); control group: 31.0 ± 17.4; (11.7–59.9)).

## 4. Discussion

Recent studies have underlined the role of oxidative stress in ED introducing the concept of penile neuropathy as a free radical dependent oxidative stress injury [34]. It is also well known that NO, derived from endothelial and neuronal sources, plays an essential role in the early phases of erection [3, 18, 35]. Therefore the interaction between nitric oxide, synthesized by eNOS, and reactive oxygen species has been proposed as one of the most important mechanisms implicated in the pathophysiological process of the ED [36].

With regard to NO production a new view has been provided by recent observations that a number of cytosolic and nuclear proteins carry single residues of O-GlcNAc and undergo functional modification depending on the presence or removal of the amino sugar [17]. In particular, eNOS is activated by phosphorylation at Ser-1177, by phosphatidylinositol-3-kinase/Akt/eNOS [37], and inactivated by removal of phosphate and addiction of O-GlcNAc residues at the same site by OGT [38]. By converse, removal of O-GlcNAc residues, catalysed by O-GlcNAcase, allows the phosphorylation of Ser-1177 and the activation of eNOS. It has been demonstrated that human RBCs express an active and functional endothelial-type eNOS, localized in both the plasma membrane and the cytoplasm [19].

Moreover, there is a growing body of evidence that an increased production of ROS leads to an increase in* O*-GlcNAcylation levels [39]; consequently, it is reasonable to hypothesize an emerging role of dysfunctional protein O-GlcNAcylation/phosphorylation in ED.

Therefore, we decided to undertake a research in a group of ED patients aimed to ascertain the possible relation between membrane-bound and cytosolic glycohydrolases content and praecox alterations due to oxidative stress.

To evaluate OS we used classical methodologies (plasma hydroperoxide levels and membrane fluidity determination) and measured antioxidant total defences by the Lag-time method [33]. This analytical approach is useful as prognostic index of plasmatic peroxidation risk in patients with oxidative pathologies (such as diabetes, cancer, hypertension, Down syndrome, and chronic renal failure) [13, 40] and is an efficient tool to monitor the effects of antioxidant treatments [25].

Our findings suggest some interesting considerations. First of all, both the evaluation of plasma hydroperoxides and Lag-time (Figure 1) confirm previous observations showing that the condition of oxidative stress in ED patients [26] overlaps the well-known OS status in patients with other pathologies [40]. Regarding peroxidation parameters, an intriguing point is the observation that in ED subjects RBC membrane fluidity shows no significant differences between patients and controls (Figure 2).

Furthermore, the activities of the assayed glycohydrolases in erythrocyte plasma membranes corroborate the evidence resulting from plasma peroxidation parameters. Indeed, the higher level of these membrane enzymes in ED patients follows a very similar pattern already observed in Down syndrome patients [13], characterised by high level of oxidative stress, and is opposite to the enzymatic pattern seen in air force acrobatic pilots, known to be in an excellent oxidative status [14]. This scenario, also considering the similar levels of fluidity membrane between ED patients and control subjects, may offer a further support of the role of these membrane glycohydrolases in signaling early membrane alterations before they become evident at a general level across the membrane and detectable using fluorescent probes, as showed in Figure 2.

Finally, the behavior of the two assayed glycohydrolases in erythrocyte cytosol, Hexosaminidase and O-GlcNAcase, undergoes a marked diminution in ED patients as compared to controls (Figure 3). This pattern is quite similar to that observed in Down syndrome patients [13] and opposite to that seen in air force acrobatic pilots [14], and it stimulates us to perform further investigations to study in depth and clarify the relationship (that, to our knowledge, is yet to be understood) between cytosolic HEX and oxidative stress.

Of particular interest is the behaviour of O-GlcNAcase. As already mentioned,* O*-GlcNAcylation plays a crucial role in the complex signalling network involved in regulating cellular responses to physiological and pathological stress stimuli [41]. We can reasonably suppose that the observed significant decrease of cytosolic O-GlcNAcase in ED patients leads to a more persistent* O*-GlcNAcylation of eNOS Ser 1177 residue, hence enforcing the hypothesis of the crucial involvement of RBC-NOS/eNOS in the mechanism of penile erection.

## 5. Conclusions

Our data give further support to the role played by oxidative stress in ED patients and emphasize the diagnostic potential of the studied RBC glycohydrolases, as early biomarkers of oxidative stress. Our data also provide an analytical support to monitor the efficacy of antioxidant therapies aimed at correcting oxidative stress [42].

Moreover, considering how, although indirectly, our data suggest a scenario of increased glycosylations, additional studies are also required to confirm and better define the role played by erythrocyte O-GlcNAcase in the inhibition of RBC-NOS activity and the subsequent failure to increase blood flow in corpora cavernosa.

## Figures and Tables

**Table 1:** Clinical and metabolic parameters of controls and ED patients.

	Controls *N* = 30	ED patients *N* = 39	Reference ranges
Age, years	50.1 ± 16.6	52.9 ± 10.6	
Glycated hemoglobin	5.0 ± 0.4	5.1 ± 0.4	≤6.5 (%)
Total cholesterol	180 ± 18	175 ± 16	<200 (mg/dL)
HDL cholesterol	42 ± 9	49 ± 10	>35 (mg/dL)
LDL cholesterol	107 ± 15	117 ± 25	<160 (mg/dL)
Triglycerides	114 ± 31	115 ± 45	<160 (mg/dL)
17-beta estradiol	38 ± 5	28 ± 8	<60 (pg/mL)
Prolactin	12 ± 4	9 ± 5	2–17 (ng/mL)
Testosterone	5.1 ± 0.9	4.6 ± 2	3–10 (ng/mL)
LH	6.9 ± 2.9	5.7 ± 2.3	1.3–13 (U/L)

HDL: high-density lipoprotein; LDL: low-density lipoprotein; LH: luteinizing hormone.

Values are expressed as mean ± standard deviation (SD).
